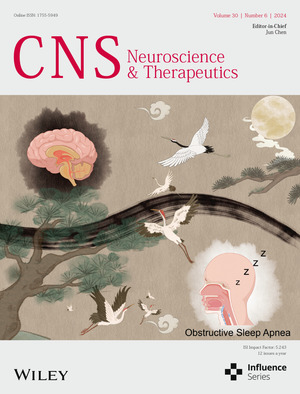# Front Cover

**DOI:** 10.1111/cns.14834

**Published:** 2024-07-02

## Abstract

The cover image is based on the Original Article *Abnormal dynamic functional connectivity and topological properties of cerebellar network in male obstructive sleep apnea* by Lifeng Li et al., https://doi.org/10.1111/cns.14786. Special thanks to Figdraw (www.figdraw.com) for their contribution to the creation of the Cover Image.